# Quantification of Fecal Glucocorticoid Metabolites in Shelter Cats Using LC/MS/MS: Potential Biomarkers of Stress Associated with Shelter Admission

**DOI:** 10.3390/ani16203227

**Published:** 2026-10-14

**Authors:** Yamato Mashimo, Yared Beyene Yohannes, Shouta M. M. Nakayama, Yoshinori Ikenaka, Mayumi Ishizuka

**Affiliations:** 1 Laboratory of Toxicology, Faculty of Veterinary Medicine, Hokkaido University, Sapporo 060-0818, Hokkaido, Japan; mashimo.yamato.m8@elms.hokudai.ac.jp (Y.M.);; 2 Department of Veterinary Forensics, Faculty of Veterinary Medicine, Hokkaido University, Sapporo 060-0818, Hokkaido, Japan; 3 School of Veterinary Medicine, The University of Zambia, P.O. Box 32379, Lusaka 10101, Zambia; 4 One Health Research Center, Hokkaido University, Sapporo 060-0818, Hokkaido, Japan; 5 Translational Research Unit, Veterinary Teaching Hospital, Faculty of Veterinary Medicine, Hokkaido University, Sapporo 060-0818, Hokkaido, Japan; 6 Water Research Group, School of Environmental Sciences and Development, North-West University, Private Bag X6001, Potchefstroom 2531, South Africa

**Keywords:** shelter cats, fecal glucocorticoid metabolites (FGMs), cortisol, environmental stress, LC/MS/MS, stress biomarkers

## Abstract

Cats can experience physiological stress during transitions to unfamiliar environments, such as shelter admission. To enable non-invasive stress assessment in cats, fecal glucocorticoid metabolites were quantified by liquid chromatography–tandem mass spectrometry (LC/MS/MS) with Girard’s reagent T derivatization. Five analytes were targeted: three glucocorticoids (cortisol, cortisone, and corticosterone) and their fecal metabolites, 11β-hydroxyetiocholanolone and 11-oxoetiocholanolone. Thirty-five cats housed at an animal welfare center were classified into a shorter-admission group (<2 weeks after admission, *n* = 16) or a longer-admission group (≥2 weeks, *n* = 19). The three parent glucocorticoids showed no detectable peak in any sample, whereas both metabolites were detected in 28 of the 35 samples and were strongly correlated in both the shorter- and longer-admission groups (ρ = 0.9316 and 0.9699, respectively). Both metabolites were significantly higher in the shorter-admission group (*p* = 0.0036 and 0.0029, respectively), whereas no significant differences were detected by sex or neuter status. In an incubation experiment, concentration changes within 48 h (≤0.12 ng/mg) were smaller than the between-group differences (1.70–2.16 ng/mg). These findings suggest that both metabolites are potential biomarkers of stress associated with shelter admission and that their measurement may provide a practical, non-invasive approach for monitoring stress in shelter cats.

## Simple Summary

Cats are sensitive to environmental changes, and objective, non-invasive methods for assessing stress are important for their welfare. In this study, we quantified fecal glucocorticoid metabolites, which are stress-related hormone metabolites excreted in feces, using liquid chromatography–tandem mass spectrometry, which enables selective quantification of trace compounds. Previous studies have reported that stress in many cats decreases over the first two weeks in a new environment. To investigate whether these metabolites reflect stress after shelter admission, we therefore compared cats sampled less than two weeks after admission with cats housed for longer periods. The results showed that cortisol, cortisone, and corticosterone had no detectable peak, whereas two of their metabolites (11β-hydroxyetiocholanolone and 11-oxoetiocholanolone) were detected, and both were significantly higher in the recently admitted cats. This association with time since admission suggests that these metabolites are potential biomarkers of stress associated with shelter admission. In addition, changes in metabolite concentrations within 48 h after defecation were smaller than the between-group differences, suggesting that fecal samples may be useful even where collection timing and temperature cannot be strictly controlled. Overall, measurement of fecal glucocorticoid metabolites may provide a practical, non-invasive approach for monitoring stress responses and welfare in shelter cats.

## 1. Introduction

Domestic cats (*Felis catus*) are known to be sensitive to environmental changes, and physical environmental changes such as relocation or transfer to a new housing facility can serve as significant stressors [1]. Shelter admission represents a substantial environmental change that may impose a stress burden on individual cats. Monitoring the adaptation of shelter cats to their new environment is therefore an important component of shelter management.

Stress responses to the shelter environment have been extensively studied in dogs, with changes in behavioral and physiological stress responses reported over the days following admission [2]. Similarly in cats, environmental change activates stress response systems such as the hypothalamic–pituitary–adrenocortical (HPA) axis and the sympathoadrenomedullary (SAM) system, eliciting physiological stress responses. HPA axis activation stimulates glucocorticoid secretion, including cortisol, from the adrenal cortex to maintain homeostasis; however, chronic activation can lead to immune suppression and increased disease risk [3].

Serum cortisol concentration is commonly measured to assess stress status; however, because blood hormone levels reflect acute fluctuations on a timescale of minutes [4], the handling required for blood collection itself can induce acute stress and alter baseline values [5]. Urinary cortisol measurement has also been attempted as a non-invasive approach and is considered to reflect stress changes on a timescale of hours [6]; however, urine collection is often technically difficult in cats because urine is typically absorbed by the litter at the time of urination [7].

Fecal sampling, by contrast, is relatively straightforward and imposes minimal burden on animals. Fecal glucocorticoid metabolites (FGMs) reflect the physiological stress status integrated over a period of one to two days [7,8] and may therefore serve as useful indicators for evaluating adaptation to the housing environment.

Enzyme immunoassay (EIA) has been widely used for FGM measurement [9]; however, overestimation due to antibody cross-reactivity has been noted [10]. In particular, non-target compounds in fecal samples containing many interfering substances may influence measured values [11,12]. Graham and Brown demonstrated that no immune reaction was detected in cat fecal metabolites using a cortisol antibody-based RIA, whereas some metabolites could be quantified using a corticosterone RIA [13]. Subsequently, Schatz and Palme compared multiple EIAs and reported that the 11-oxoetiocholanolone EIA showed the highest immunoreactivity in cat feces [7]. Meanwhile, EIA using cortisol antibodies continues to be used for FGM measurement in felids [14,15,16], and the optimal antibody has not been clearly established; further investigation is therefore needed to identify the specific molecules being measured.

Recent advances in mass spectrometry have enabled quantitative determination of specific steroid molecules with high selectivity and sensitivity using LC/MS/MS [17]. However, when applied to samples with high matrix complexity such as feces, matrix effects can suppress the ionization of trace neutral steroids [18]. Derivatization with Girard’s reagent T (GRT), which improves detection sensitivity in mass spectrometry by introducing a positive charge onto carbonyl-containing molecules, was therefore adopted [19,20,21].

In the present study, we hypothesized that environmental changes following shelter admission induce stress, and cats in a shelter environment were classified into a shorter-admission group (<2 weeks after admission) and a longer-admission group (≥2 weeks after admission). GRT-derivatized fecal samples were analyzed by LC/MS/MS to quantify the parent glucocorticoids cortisol, cortisone, and corticosterone, together with their metabolites 11β-hydroxyetiocholanolone and 11-oxoetiocholanolone [7,22], with the aim of quantitatively evaluating stress in shelter cats and examining the utility of each analyte as a candidate stress biomarker for non-invasive stress assessment in this population.

## 2. Materials and Methods

### 2.1. Animals and Housing Conditions

This prospective observational study was conducted at a municipal animal welfare center. The subjects were 35 cats housed at the facility, admitted following hoarding cases, owner surrenders, or rescue as strays. All cats were housed individually in cages (approximately 93 cm wide × 63 cm deep × 121 cm high). Human contact was limited to daily feeding and cleaning procedures, as well as observation by facility visitors. The ambient temperature and relative humidity were maintained at 24 °C and 50%, respectively. Feeding was provided twice daily at 09:00 and 16:00. Cats adopted during the study period were excluded from further analyses following adoption. During the sampling period, all cats were fed the same commercial cat food in accordance with the routine feeding management of the shelter. Cats with physical findings such as obvious trauma or renal failure and cats undergoing treatment for disease were not included in this study. Diarrheic feces were not included in the analysis in order to standardize fecal consistency.

For group classification, criteria were established based on findings from previous studies. Kessler and Turner reported that behavioral stress in cats housed in boarding catteries decreased over two weeks, with approximately two-thirds of the cats reaching low stress levels [23]. Kry and Casey also reported that in shelter cats provided with enrichment, stress continued to decrease until 14 days after admission [24]. In dogs, plasma cortisol concentrations during the first three days in the shelter were reported to be higher than those in dogs housed for more than nine days, with intermediate levels on days 4–9 [25].

Based on these findings, subjects were classified into the following two groups. Details of individual animals are provided in Supplementary Table S1.

Shorter-admission group: Individuals less than two weeks after admission (*n* = 16), defined as the period during which stress responses associated with environmental change were expected to be present.

Longer-admission group: Individuals two weeks or more after admission (*n* = 19), defined as the period during which stress responses associated with environmental change were expected to have decreased.

### 2.2. Sample Collection

Fecal samples were collected between 08:00 and 09:00 the following morning from material defecated during the facility’s closed hours (17:00–08:00 the next day). Samples were immediately frozen after collection and stored at −80 °C or below until analysis. At the time of analysis, samples were freeze-dried under vacuum, and 20 mg of dried feces was used as the analytical sample.

### 2.3. Stability of Fecal Steroid Metabolites After Defecation

To evaluate the effects of ambient temperature and elapsed time after defecation on fecal steroid metabolite concentrations, an incubation experiment was conducted. Fecal samples collected from three individuals within 1 h after defecation were divided into the following conditions: immediately after collection (0 h), incubated at 20 °C (room temperature) for 24 h and 48 h, and incubated at 40 °C (incubator) for 24 h and 48 h. After each designated time period, samples were promptly frozen at −80 °C to suppress further metabolism in the feces. Samples were then freeze-dried, and a pooled sample was prepared for each condition by mixing equal weights of feces from the three individuals in order to average out inter-individual variation. Four samples of 20 mg dried feces were taken from each pooled sample as analytical replicates (*n* = 4) and subjected to analysis. Values from the incubation experiment are expressed as the mean ± standard deviation of the four analytical replicates.

### 2.4. Sample Preparation and Derivatization

Approximately 20 mg of dried feces was placed in the Micro Volume QuEChERS Kit (Shimadzu Corporation, Kyoto, Japan), and 380 µL of acetonitrile and 300 µL of distilled water were added. At the start of extraction, 50 µL of deuterium-labeled internal standards (IS; 200 ng/mL) was added. After mixing, the samples were centrifuged (10,000× *g*, 10 min, 4 °C), and 300 µL of the organic layer was obtained. Details of the IS used and their assignment to each target compound are provided in Supplementary Table S2.

To introduce cationic functional groups at the ketone moieties of steroid molecules, 50 µL of Girard’s reagent T (GRT) (Tokyo Chemical Industry Co., Ltd., Tokyo, Japan) solution (100 mM GRT/0.4 M acetate buffer, pH 2.5) was added to the organic layer obtained by the QuEChERS extraction, and the mixture was heated at 70 °C for 20 min using a block heater [21].

After the reaction, 350 µL of the reaction mixture was applied to a solid-phase extraction (SPE) column (EVOLUTE EXPRESS CX 30 mg/1 mL, Biotage, Uppsala, Sweden). The column was washed with 500 µL of acetonitrile, and the resulting 850 µL of eluate was concentrated to dryness using a centrifugal evaporator. The residue was reconstituted to 250 µL with 30% methanol containing 0.1% formic acid to prepare the LC/MS/MS analytical sample.

### 2.5. LC/MS/MS Analysis

Analyses were performed using a triple quadrupole mass spectrometer Agilent Ultivo (Agilent Technologies, Santa Clara, CA, USA) operated with electrospray ionization in positive ion mode and coupled to an Agilent 1290 Infinity II LC system. A Poroshell 120 EC-C18 column (2.7 µm, 3.0 × 100 mm, Agilent Technologies) was used, with the column temperature set at 45 °C.

The mobile phases consisted of (A) 0.1% formic acid in water and (B) 0.1% formic acid in methanol. Detailed gradient conditions are provided in Supplementary Table S3.

Five analytes were targeted: the parent compounds cortisol, cortisone, and corticosterone, and the metabolites 11β-hydroxyetiocholanolone and 11-oxoetiocholanolone. Detection was performed using multiple reaction monitoring (MRM) mode, and quantification was performed using Agilent MassHunter Quantitative Analysis software (version 12.0, Agilent Technologies). MRM transitions, collision energies (CE), and other mass spectrometry parameters for each compound are provided in Supplementary Table S4.

The limit of detection (LOD) was 1.25 pg/mg for all five analytes, and the method quantification limit (MQL) was 125 pg/mg for the three parent glucocorticoids. The MQL could not be determined for the two metabolites, which are endogenously present in feces and for which a blank fecal matrix was therefore unavailable. Matrix effects and absolute recovery are provided in Supplementary Table S5.

### 2.6. Statistical Analysis

All statistical analyses were performed using statistical software (JMP Pro version 19.1.0, SAS Institute Inc., Cary, NC, USA). Normality was assessed using the Shapiro–Wilk test, and as the data did not follow a normal distribution, non-parametric tests were applied. Measured values of samples in which no peak was detected were treated as 0 (a value of 0 indicates no detectable peak, not the concentration in feces) and were included in all analyses, including the group comparisons, correlation analyses, calculation of means and medians, and graphical presentations. Because all statistical tests were rank-based, samples with no detectable peak were treated as tied at the lowest rank. Differences in steroid concentrations between the shorter- and longer-admission groups were assessed using the Wilcoxon rank-sum test. Comparisons by sex and neuter status were performed using the Steel-Dwass test. Spearman’s rank correlation coefficients were calculated to assess correlations between steroid metabolites within the shorter- and longer-admission groups separately, as well as for all cats combined. A two-sided *p*-value of less than 0.05 was considered statistically significant for all tests.

## 3. Results

### 3.1. Study Population

A total of 35 cats were included in this study (shorter-admission group, *n* = 16; longer-admission group, *n* = 19). One additional cat was excluded from the analyses because blindness was identified after sampling. At the time of sampling, the median number of days from admission was 4 days (range, 1–12) in the shorter-admission group and 48 days (range, 14–344) in the longer-admission group. The distribution of intake categories in each group is shown in Table 1.

### 3.2. Fecal Steroid Profiling

**Figure 1:**
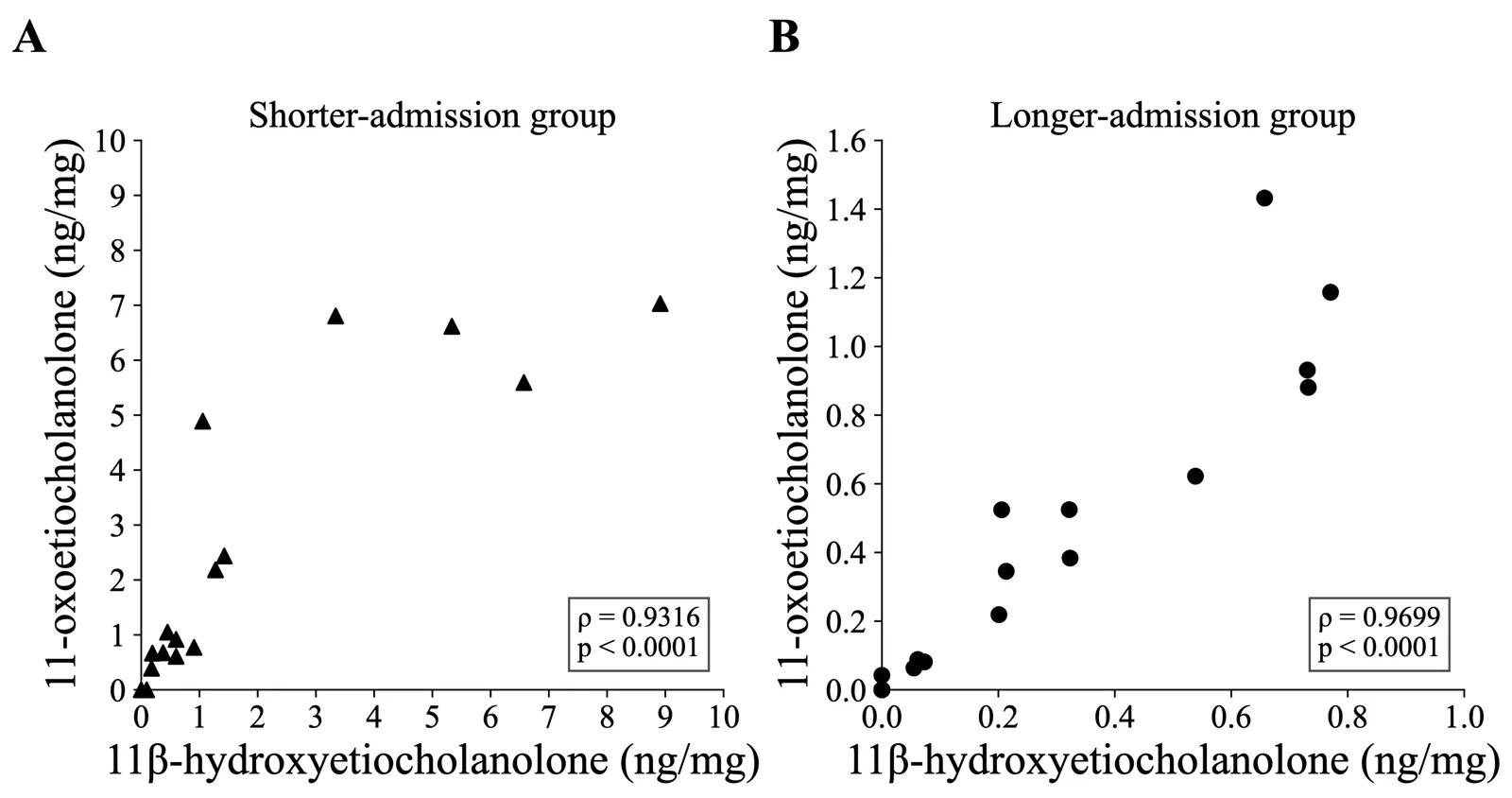
Correlation between fecal 11β-hydroxyetiocholanolone and 11-oxoetiocholanolone concentrations within each group. (**A**) Shorter-admission group (*n* = 16; ρ = 0.9316, *p* < 0.0001). (**B**) Longer-admission group (*n* = 19; ρ = 0.9699, *p* < 0.0001). Each data point represents an individual cat. Correlations were assessed using Spearman’s rank correlation coefficient.

The major metabolites quantified in feces, 11β-hydroxyetiocholanolone and 11-oxoetiocholanolone, were each detected in 28 of the 35 samples (11β-hydroxyetiocholanolone: 13/19 in the longer-admission group and 15/16 in the shorter-admission group; 11-oxoetiocholanolone: 14/19 and 14/16, respectively), and a strong positive correlation was observed between these two compounds in both the shorter-admission group (*n* = 16, ρ = 0.9316, *p* < 0.0001; Figure 1A) and the longer-admission group (*n* = 19, ρ = 0.9699, *p* < 0.0001; Figure 1B). A comparable correlation was also observed when all cats were analyzed together (*n* = 35, ρ = 0.9568, *p* < 0.0001). In contrast, the parent compounds cortisol, cortisone, and corticosterone showed no detectable peak in any of the 35 samples.

### 3.3. Comparison by Length of Admission

**Figure 2:**
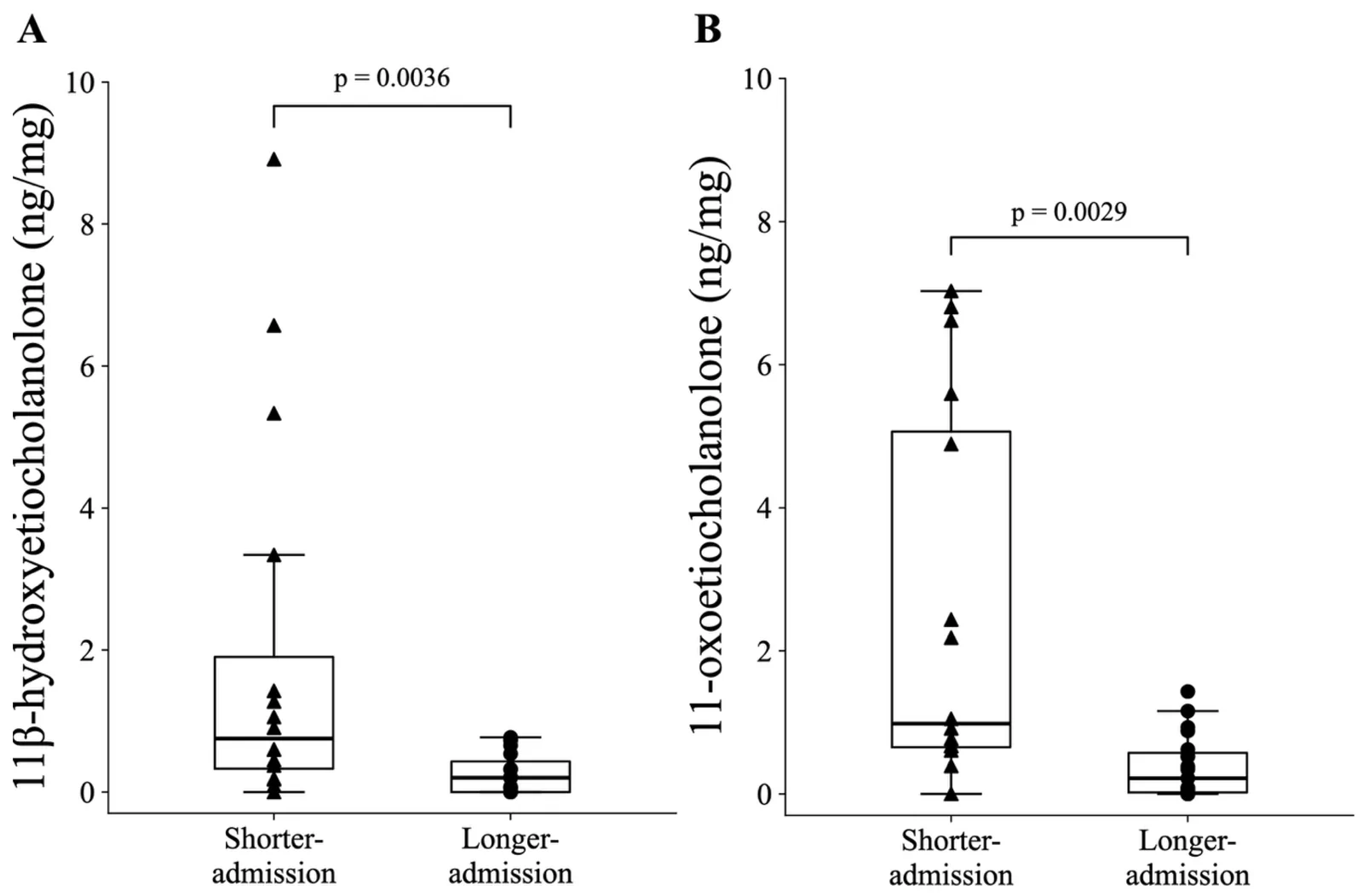
Comparison of fecal glucocorticoid metabolite concentrations between the shorter- and longer-admission groups. (**A**) 11β-hydroxyetiocholanolone, (**B**) 11-oxoetiocholanolone. Box plots show the median, interquartile range, and 1.5× interquartile range. Individual data points are overlaid (longer-admission group: *n* = 19, shorter-admission group: *n* = 16). Group comparisons were performed using the Wilcoxon rank-sum test: (**A**) *p* = 0.0036; (**B**) *p* = 0.0029.

In the comparison between the longer-admission group (≥2 weeks after admission) and the shorter-admission group (<2 weeks after admission), both 11β-hydroxyetiocholanolone and 11-oxoetiocholanolone were significantly higher in the shorter-admission group (*p* = 0.0036 and *p* = 0.0029, respectively; Figure 2). For 11β-hydroxyetiocholanolone, concentrations ranged from 0 (no detectable peak) to 0.77 ng/mg (mean, 0.257 ng/mg; median, 0.201 ng/mg) in the longer-admission group and from 0 to 8.91 ng/mg (mean, 1.957 ng/mg; median, 0.753 ng/mg) in the shorter-admission group. For 11-oxoetiocholanolone, concentrations ranged from 0 to 1.43 ng/mg (mean, 0.384 ng/mg; median, 0.219 ng/mg) in the longer-admission group and from 0 to 7.03 ng/mg (mean, 2.540 ng/mg; median, 0.983 ng/mg) in the shorter-admission group. In the shorter-admission group, concentrations exceeding the range of the longer-admission group were observed in cats sampled up to six days after admission, whereas those in cats sampled seven days or more after admission were within the range of the longer-admission group.

### 3.4. Effects of Sex and Neuter Status

**Figure 3:**
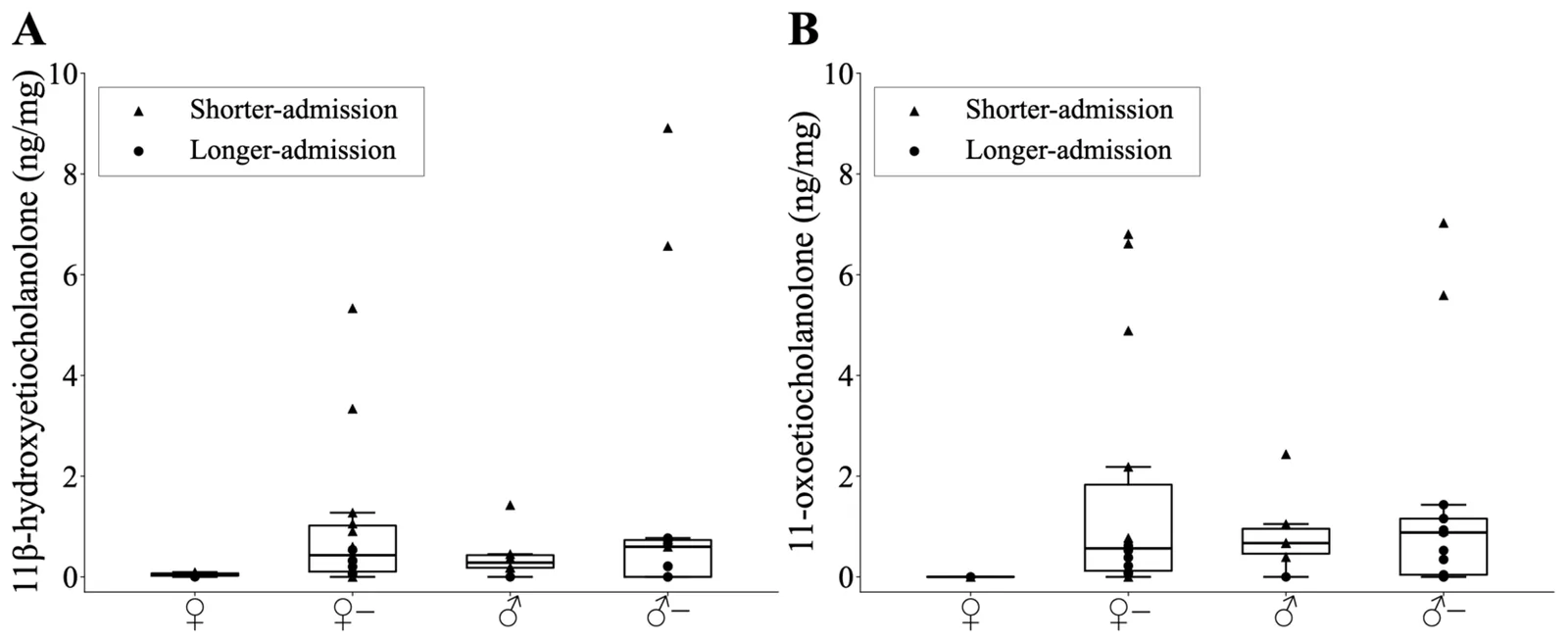
Comparison of fecal glucocorticoid metabolite concentrations among subgroups classified by sex and neuter status. (**A**) 11β-hydroxyetiocholanolone, (**B**) 11-oxoetiocholanolone. Box plots show the median, interquartile range, and 1.5× interquartile range for each subgroup (intact female: n = 2, spayed female: *n* = 14, intact male: *n* = 6, neutered male: *n* = 13). Individual data points are overlaid, with filled circles (●) indicating individuals in the longer-admission group and filled triangles (▲) indicating individuals in the shorter-admission group. Multiple comparisons were performed using the Steel-Dwass test.

Comparisons by sex and neuter status (intact female, spayed female, intact male, neutered male) detected no significant differences in concentrations of either 11β-hydroxyetiocholanolone or 11-oxoetiocholanolone (*p* > 0.05; Figure 3).

### 3.5. Temporal Stability of Fecal Steroid Metabolites

**Figure 4:**
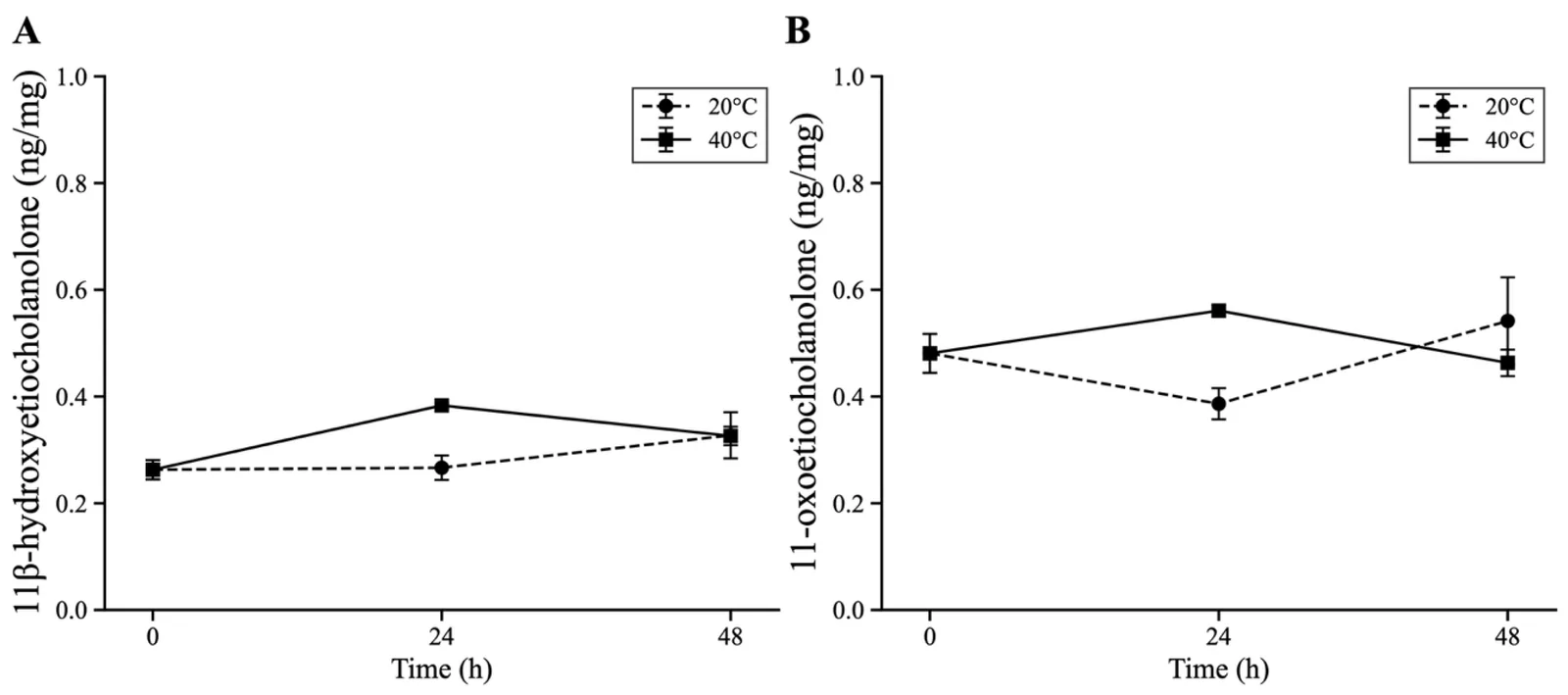
Temporal changes in fecal 11β-hydroxyetiocholanolone and 11-oxoetiocholanolone concentrations under two temperature conditions. Fecal concentrations of 11β-hydroxyetiocholanolone (**A**) and 11-oxoetiocholanolone (**B**) were measured at 0, 24, and 48 h under incubation conditions of 20 °C and 40 °C (*n* = 4 analytical replicates per condition). Data are presented as mean ± standard deviation.

The temporal changes in 11β-hydroxyetiocholanolone and 11-oxoetiocholanolone concentrations during incubation at 20 °C (room temperature) and 40 °C are shown in Figure 4. Mean concentrations immediately after defecation (0 h) were 0.26 ng/mg for 11β-hydroxyetiocholanolone and 0.48 ng/mg for 11-oxoetiocholanolone. Across all time points (0, 24, and 48 h) and both temperature conditions, mean concentrations ranged from 0.26 to 0.38 ng/mg for 11β-hydroxyetiocholanolone and from 0.39 to 0.56 ng/mg for 11-oxoetiocholanolone.

## 4. Discussion

The shorter-admission group (<2 weeks after admission) showed significantly higher FGM concentrations than the longer-admission group, with 11β-hydroxyetiocholanolone ranging from 0 to 8.91 ng/mg and 11-oxoetiocholanolone ranging from 0 to 7.03 ng/mg, indicating a wide distribution. Kessler and Turner behaviorally assessed 140 cats during a two-week stay in boarding catteries using the Cat Stress Score and reported that stress declined markedly during the first four days and continued to decrease over the two weeks, approaching the levels of cats established in a shelter, with approximately two-thirds of the cats showing low stress levels [23]. Similarly, Kry and Casey reported that in cats housed in a shelter with enrichment, stress levels decreased significantly through day 5 after admission and continued to decrease between days 5 and 14 [24]. In the shorter-admission group of the present study, concentrations exceeding the range of the longer-admission group were observed in cats sampled up to six days after admission, whereas those in cats sampled seven days or more after admission were within the range of the longer-admission group. As fecal glucocorticoid metabolites reflect the physiological state over the preceding one to two days [7,8,13], the high values up to day six may have captured the period during which behavioral stress was decreasing but remained high, whereas the low values from day seven onward may have captured the period during which behavioral stress had largely stabilized. The wide concentration distribution observed in the shorter-admission group may also reflect individual differences in the capacity to cope with stress associated with environmental change to an unfamiliar environment [26,27,28]. However, as this study was cross-sectional, longitudinal studies are needed to clarify temporal changes after admission.

The two FGMs found to differ significantly between the shorter- and longer-admission groups—11β-hydroxyetiocholanolone and 11-oxoetiocholanolone—are both metabolites derived primarily from glucocorticoids. In experiments targeting urinary glucocorticoid metabolites in humans, cortisol administration to patients with 17-hydroxylase deficiency, who lack adrenal androgen production capacity, resulted in urinary excretion of both compounds equivalent to that in healthy individuals, demonstrating that almost all of these compounds are derived from the glucocorticoid pathway [29]. Furthermore, the strong correlation observed between the two compounds in both the shorter- and longer-admission groups (ρ = 0.9316 and 0.9699, respectively) indicates that their dynamics are similar. Based on these findings, the concentrations of the two FGMs identified in this study are suggested to primarily reflect glucocorticoid metabolism. However, as these findings were obtained in humans, detailed metabolic studies in cats are needed.

Additionally, a study monitoring cortisol metabolism in dogs and cats reported that 81.9 ± 3.8% (mean ± SD) of metabolites derived from administered cortisol were excreted via feces in cats, whereas 76.6 ± 4.1% were excreted via urine in dogs [7], demonstrating a marked difference in cortisol excretion routes between the two species. A study evaluating steroid hormone metabolism in cats using bile duct cannulas also reported that approximately 70–90% of cortisone metabolites were excreted via the biliary route [30]. While urinary cortisol has been widely used as a non-invasive stress biomarker in dogs [31,32,33,34], feces represent the primary excretion route in cats, suggesting that measurement of fecal metabolites is more appropriate for this species. Despite high-sensitivity analysis, the parent compounds (cortisol, cortisone, and corticosterone) showed no detectable peak in cat feces. As noted above, while 81.9% of FGMs are excreted via feces in cats, cortisol itself is rarely detected in feces [7]. The absence of parent compounds in this study is consistent with this finding. Therefore, when assessing stress in cats using fecal glucocorticoids, targeting metabolites rather than parent compounds is considered more appropriate.

Ramos et al. quantified FGM using an 11-oxoetiocholanolone EIA and reported mean FGM concentrations of 0.268–0.338 ng/mg dry across housing groups in domestic cats housed in private households with no obvious behavioral problems [35]. Although concentrations measured by EIA depend on the cross-reactivity of the antibody and cannot be directly compared with those measured by LC/MS/MS [8], the 11-oxoetiocholanolone concentration in the longer-admission group of the present study (mean, 0.384 ng/mg dry) was within a similar range. In addition, Ellis et al. tracked the same individually caged cats over a 30-day period using an 11-oxoetiocholanolone EIA and reported that FGM concentrations peaked at a mean of 0.355 ng/mg in week 1 and declined significantly to a mean of 0.096 ng/mg by week 5 [27]. In the present study, cats within approximately two weeks of admission (shorter-admission group) had higher FGM concentrations than those housed for two weeks or longer (longer-admission group). Higher concentrations in the early period after admission were consistent with the findings of Ellis et al. However, whereas Ellis et al. monitored the same individuals longitudinally, the present study was a cross-sectional comparison of groups defined by length of stay, because many cats were adopted at an early stage and could not be tracked over an extended period. Longitudinal monitoring of individual cats is required to determine whether FGM concentrations similarly decline with continued shelter residence.

In the fecal incubation experiment, FGM concentrations were found to vary over time under both 20 °C and 40 °C conditions. This variation may be attributable to the enzymatic activity of bacteria present in feces after defecation; changes in glucocorticoid metabolites caused by fecal bacteria have been reported previously [36,37,38]. However, the variation was limited to a maximum of approximately 0.12 ng/mg for 11β-hydroxyetiocholanolone and approximately 0.09 ng/mg for 11-oxoetiocholanolone. In contrast, the mean intergroup concentration difference was approximately 1.70 ng/mg for 11β-hydroxyetiocholanolone and 2.16 ng/mg for 11-oxoetiocholanolone, exceeding the maximum temporal variation observed during incubation. However, this experiment was based on analytical replicates of a pooled sample derived from three individuals, and further verification is required before the temporal stability observed here can be generalized. Nevertheless, the present results suggest that FGM measurement may be applicable even in settings where strict control of collection timing and temperature is difficult. The 20 °C condition corresponds to a temperature typical of indoor housing environments, whereas the 40 °C condition represents an upper limit assumed for environments in which temperature is not controlled by air conditioning. The small temporal changes observed under both conditions may extend the range of settings in which fecal samples can be applied.

No significant differences in FGM concentrations were detected based on sex or neuter status in this study. Previous studies measuring FGMs in domestic cats housed individually or in groups also found no sex differences [35], and similarly, no sex differences were observed in studies using urinary cortisol as an indicator [6,39,40]. Research monitoring glucocorticoid metabolites in humans has indicated that both compounds are derived predominantly from glucocorticoid metabolism, with minimal contribution from androgen metabolism [29]. If androgen metabolism does not influence the formation of these two metabolites in cats, this may partly explain the absence of detected sex differences. However, reports exist of higher FGM concentrations in intact male domestic cats compared with other groups [13], and the involvement of gonadal hormones cannot be entirely excluded. Furthermore, a comprehensive review of FGMs in mammals has noted sex differences in FGM concentrations across multiple species including domestic cats [4]. The sample size in this study was limited, and the possibility of a type II error due to the small subgroup sizes cannot be excluded; further investigation into sex differences is needed.

### Limitations

This study has several limitations. First, factors other than adrenocortical activity that may affect fecal glucocorticoid metabolite concentrations include body weight, as well as diet, fecal bulk, and defecation frequency [8]. To minimize their influence, all cats were fed the same commercial diet and diarrheic feces were excluded; however, body weight could not be measured in all cats because some individuals were extremely difficult to handle. In addition, the influence of individual differences in factors such as fecal output and gastrointestinal transit time cannot be excluded.

Secondly, many of the shelter cats included in this study were of unknown exact age, with estimated ages derived from physical examination. Consequently, the relationship between age and the concentration of FGMs or stress coping capacity could not be evaluated.

Thirdly, for the shorter-admission group, the possibility that pre-rescue dietary history influenced the data cannot be excluded; differences in nutritional status resulting from feeding conditions may have affected glucocorticoid metabolism and secretion.

Fourthly, the stressors to which individual cats had been exposed prior to rescue were unknown. The study population included cats with diverse backgrounds, such as those rescued from hoarding situations and strays, making it difficult to determine the nature and duration of pre-rescue stressor exposure. Further validation is needed with more comprehensive background information, as well as accurate characterization of sampling conditions and dietary and nutritional status.

Fifthly, the present study was conducted at a single shelter facility. Although this contributes to the stability of the data by homogenizing individual conditions, further verification is required at other facilities to confirm whether comparable results are obtained and to establish the utility of the identified FGMs as stress biomarkers.

Finally, this study employed a cross-sectional design and could not track within-individual changes over time. The classification into the shorter- and longer-admission groups was based solely on time since admission, and no behavioral or other indicators of stress or acclimation independent of FGM were assessed. Accordingly, the difference in FGM concentrations between the two groups indicates an association with time since admission, and establishing the validity of these metabolites as indicators of acclimation to the shelter environment will require longitudinal sampling of the same individuals together with assessment using other measures, such as behavioral evaluation.

## 5. Conclusions

This study suggests that 11β-hydroxyetiocholanolone and 11-oxoetiocholanolone, quantified in fecal samples by LC/MS/MS, are potential biomarkers of stress associated with shelter admission. Both metabolites were detected, whereas the three parent glucocorticoids (cortisol, cortisone, and corticosterone) showed no detectable peak in any sample. Significantly higher FGM concentrations were observed in the shorter-admission group (<2 weeks after admission) compared with the longer-admission group (≥2 weeks after admission), indicating that FGM levels are associated with the time since shelter admission. Therefore, LC/MS/MS-based fecal FGM measurement may provide a practical and non-invasive approach for monitoring stress responses and welfare assessment in shelter cats. Combining FGM measurement with other hormonal and behavioral indicators may allow stress to be assessed from multiple perspectives.

## Figures and Tables

**Table 1:** Characteristics of the cats in each group.

Characteristic	Shorter-Admission (*n* = 16)	Longer-Admission (*n* = 19)
Median days from admission	4 (1–12)	48 (14–344)
Intake category (*n*)		
Hoarding case	8	9
Owner-surrendered	8	6
Stray	0	4

## Data Availability

The data presented in this study are available in the Supplementary Materials. Further data are available from the corresponding author upon reasonable request.

## Supplementary Materials

The following supporting information can be downloaded at https://www.mdpi.com/article/10.3390/ani16203227/s1, Table S1: Individual information of cats included in this study; Table S2: Standard solution and internal standard solution used in this study; Table S3: Mobile phase, flow rate, column temperature, and mobile phase gradient conditions; Table S4: Mass spectrometry and multiple reaction monitoring (MRM) conditions; Table S5: Limit of Detection (LOD), Method Quantification Limit (MQL), absolute recovery, and matrix effects of target analytes.
