# Supplementary material for: Quantification of Fecal Glucocorticoid Metabolites in Shelter Cats Using LC/MS/MS: Potential Biomarkers of Stress Associated with Shelter Admission
Source: Animals (Basel). 2026 Oct 14;16(20):3227. doi: 10.3390/ani16203227 (PMC13700595; doi:10.3390/ani16203227)
Supplement: Supplementary file 1 [file animals-16-03227-s001.zip › Supplementary_Tables_S2_S3_R1.pdf]

Table S2. Standard solution and internal standard solution used in this study.

Standard solution		Internal standard solution	
**Chemicals**	**Manufacturer**	**Chemicals**	**Manufacturer**
11β-hydroxyetiocholanolone	Cayman Chemical (Ann Arbor, MI, USA)	11-oxoetiocholanolone-D5	MedChemExpress (Monmouth Junction, NJ, USA)
11-oxoetiocholanolone	Cayman Chemical (Ann Arbor, MI, USA)		
Corticosterone	Cerilliant (Round Rock, TX, USA)	GT-Corticosterone-D4	Cerilliant (Round Rock, TX, USA)
Cortisol	Cerilliant (Round Rock, TX, USA)	GT-Cortisol-D4	Cerilliant (Round Rock, TX, USA)
Cortisone	Cerilliant (Round Rock, TX, USA)	GT-Cortisone-D8	Toronto Research Chemicals (Toronto, ON, Canada)

11-oxoetiocholanolone-D5 was used as the internal standard for both 11β-hydroxyetiocholanolone and 11-oxoetiocholanolone because of their structural similarity and because a deuterium-labeled 11β-hydroxyetiocholanolone was not readily available commercially.

Table S3. Mobile phase, flow rate, column temperature, and mobile phase gradient conditions.

Mobile phase A	Water containing 0.1% formic acid
Mobile phase B	Methanol containing 0.1% formic acid
Flow rate	0.6 mL/min
Column temperature	45 °C

Gradient condition

Time (min)	Mobile phase A (%)	Mobile phase B (%)	Flow rate (mL/min)
0 min (start)	90	10	0.600
0.50	90	10	—
12.00	0	100	—
14.00	0	100	—
14.01	90	10	—
