# Supplementary material for: Quantification of Fecal Glucocorticoid Metabolites in Shelter Cats Using LC/MS/MS: Potential Biomarkers of Stress Associated with Shelter Admission
Source: Animals (Basel). 2026 Oct 14;16(20):3227. doi: 10.3390/ani16203227 (PMC13700595; doi:10.3390/ani16203227)
Supplement: Supplementary file 1 [file animals-16-03227-s001.zip › Supplementary_Table S5_R1.pdf]

Supplementary Table S5. Limit of Detection (LOD), Method Quantification Limit (MQL), absolute recovery, and matrix effects of target analytes.

Compound	LOD (pg/mg)	MQL (pg/mg)	Absolute recovery (%)	Matrix effect (%)
11β-hydroxyetiocholanolone	1.25	Not determinable	51.8	33.4
11-oxoetiocholanolone	1.25	Not determinable	59.7	55.9
Cortisol	1.25	125	30.6	80.6
Cortisone	1.25	125	32.6	106.6
Corticosterone	1.25	125	31.3	82.4

LOD: The lowest standard solution concentration showing a signal-to-noise ratio (S/N) ≥ 3, divided by the fecal weight (20 mg).

MQL: The concentration of standard solution subjected to the same pre-treatment procedure as samples and meeting S/N ≥ 10, divided by the fecal weight (20 mg).

Absolute recovery (%) = (mean peak area of pre-treated standard / mean peak area of non-pre-treated standard) × 100

Matrix effect (%) = (mean peak area of pre-treated standard spiked into fecal matrix − mean peak area of endogenous fecal sample) / mean peak area of pre-treated standard × 100

Not determinable: As the analyte is endogenously present in feces, a blank fecal matrix could not be obtained and MQL could not be calculated.
